# Morphological Correlates of Suppressed Autonomic Modulation: A Machine Learning Approach to Identifying Vagal Impairment Phenotypes in Women

**DOI:** 10.3390/jfmk11030355

**Published:** 2026-09-06

**Authors:** Wollner Materko, Gustavo Ferreira das Chagas, Paulo Roberto Benchimol-Barbosa, Jurandir Nadal

**Affiliations:** 1 Biomedical Engineering Program (PEB), COPPE Institute, Universidade Federal do Rio de Janeiro, P.O. Box 68510, Rio de Janeiro 21941-972, RJ, Brazil; wollner.materko@gmail.com (W.M.); gustavo.chagas@peb.ufrj.br (G.F.d.C.); 2 Pedro Ernesto University Hospital, Universidade do Estado do Rio de Janeiro, Rio de Janeiro 20551-030, RJ, Brazil; ecgar@yahoo.com

**Keywords:** heart rate variability, central adiposity, kinanthropometry, machine learning, women’s health

## Abstract

**Background/Objectives:** Cardiac autonomic modulation, as assessed by heart rate variability (HRV), is a key indicator of physiological status. Although aging has traditionally been associated with reduced variability, morphological characteristics may be its main correlates. This study aimed to identify phenotypes of suppressed vagal modulation in women using a machine learning approach, specifically by correcting for HRV’s intrinsic mathematical dependence on heart rate (HR). **Methods:** Seventy-five women (30–69 years old) were recruited during fitness center enrollment. To control for hormonal fluctuations, the younger cohort (30–49 years old) was assessed during the follicular phase, whereas the older cohort (50–69 years old) was postmenopausal. Electrocardiogram data for HRV, bioimpedance, and anthropometric measurements were collected. Intrinsic vagal modulation was isolated by adjusting root mean square of successive differences (RMSSD) for the mean R–R interval (RMSSD_adj). The machine learning pipeline used LASSO for feature selection and multivariate logistic regression to identify suppressed vagal phenotypes (RMSSD_adj ≤ 0.0232). Model stability was verified using 1000 bootstrap iterations, and a cumulative Z-score index (ISCA) was used to characterize the morphologic–hemodynamic burden. **Results:** LASSO identified central adiposity, measured by the waist-to-hip ratio, as the primary independent correlate of suppressed vagal phenotypes (RMSSD_adj ≤ 0.0232). The model achieved an area under the curve (AUC)–ROC of 0.633 (95% CI: 0.490–0.771) with high specificity (0.900) and sensitivity of 0.360. No significant differences in intrinsic vagal modulation were observed between age cohorts (*p* > 0.05). However, women classified as having a high morphologic–hemodynamic burden (high-overload ISCA) exhibited a significantly higher resting heart rate, averaging 8.3 bpm more than the low-overload group (*p* < 0.05), reflecting a higher physiological demand in this phenotype. **Conclusions:** The autonomic status of women is characterized more accurately by morphological phenotypes than by chronological age. Integrated kinanthropometric monitoring is essential for identifying reduced autonomic resilience regardless of birth year.

## 1. Introduction

Cardiac autonomic modulation, as assessed by heart rate variability (HRV), is a key marker of physiological adaptability [1] and cardiometabolic health [2,3]. In kinesiology and functional morphology, HRV, particularly vagally mediated indices such as the root mean square of successive differences (RMSSD), serves as a proxy for neurocardiac integrity and the body’s capacity to respond to both internal and external stressors [4,5]. Reductions in these functional metrics are consistently associated with increased allostatic load, chronic inflammation, and a greater prevalence of obesity-related metabolic disorders [6,7].

In women, aging is often accompanied by considerable morphological changes, including an increase in overall adiposity and a redistribution of fat toward the visceral compartment [8]. Although chronological age is traditionally viewed as the primary cause of autonomic decline, recent kinesiological studies suggest that morphological characteristics, especially central adiposity and body composition, are the true determinants of cardiac health [9,10,11]. Anthropometric measures, such as waist circumference (WC) and visceral fat percentage, serve as functional indicators of metabolic stress that directly affect the autonomic vagal brake [12,13].

Although morphological markers are clinically significant, the specific adipose thresholds that trigger systemic autonomic impairment remain unclear. In the context of Advances in Kinanthropometry, this study connects structural morphology with autonomic associations by identifying distinct vagal association phenotypes in women transitioning from middle to older age. The study validates accessible, noninvasive markers, particularly central adiposity, as reliable proxies for neurocardiac integrity and allostatic load. This addresses a critical public health need to mitigate noncommunicable diseases. Furthermore, this work demonstrates the evolution of kinanthropometry from descriptive monitoring to accurate diagnostic assessment through the integration of machine learning and predictive modeling. This approach provides clinicians with a practical framework for tailoring interventions and aligning morphological structure with physiological health outcomes in aging populations.

This study aims to characterize vagal association phenotypes across two age groups (30–49 and 50–69 years) to better understand the relationship between functional morphology and neurocardiac modulation. Using a machine learning pipeline, we integrated anthropometric indices, bioimpedance-derived body composition, and high-resolution HRV analysis to identify the main morphological predictors of RMSSD-defined association. We hypothesized that central adiposity and visceral morphological traits would emerge as stronger drivers of autonomic dysregulation than chronological age. This would provide a validated tool for personalized kinesiological assessments and for stratifying cardiometabolic associations.

## 2. Materials and Methods

### 2.1. Study Design and Population

This retrospective, cross-sectional study used data from 75 adult women (aged 30–69 years) living in Rio de Janeiro, Brazil. The data were originally collected during routine kinanthropometric and physiological screenings performed at a local fitness center over a two-year period (January 2025 to May 2026).

The study was conducted as part of the project titled “Development of a portable six-channel wireless electrocardiograph for real-time detection of adverse cardiovascular events using artificial intelligence, edge computing and the Internet of Things,” approved by the Research Ethics Committee of Hospital Universitário Pedro Ernesto, Rio de Janeiro State University (CEP-HUPE/UERJ). Ethical oversight was managed in two administrative phases: initial approval was granted on 11 June 2026 (No. 8.502.042), authorizing the retrospective analysis of secondary data, while a subsequent administrative update was issued on 16 July 2026 (No. 8.591.352; CAAE 97984626.9.0000.5259) to finalize the wording of the institutional consent documents and conducted in strict accordance with the Declaration of Helsinki and Resolution 510/2016 of the Brazilian National Health Council.

While the broader protocol encompasses the prospective development and validation of the device, the present work focuses specifically on the retrospective analysis of a pre-existing database for calibration and quality assessment. Verbal informed consent was obtained from the participants. The rationale for utilizing verbal consent is that the study involved a retrospective analysis of secondary data collected during routine assessments.

To reduce the impact of hormonal fluctuations on HRV, women aged 30–49 were evaluated only during the early follicular phase of the menstrual cycle. This phase is characterized by low levels of estrogen and progesterone. For the older cohort (ages 50–69), participants were required to be postmenopausal, defined as having had no menstrual periods for at least 12 consecutive months.

The exclusion criteria were as follows: (1) self-reported acute illness, (2) pre-existing chronic conditions such as diabetes mellitus or diagnosed hypertension, (3) musculoskeletal injuries, (4) the use of orthopedic prostheses or cardiac pacemakers, (5) the use of ergogenic supplements, and (6) current pregnancy or menstruation.

To ensure data reliability and isolate the influence of morphological structure on cardiac autonomic modulation, testing conditions were rigorously standardized to control for acute metabolic and endocrine confounders. Participants were instructed to maintain their usual sleep schedule and avoid strenuous physical activity for 24 h before the assessment. On the day of testing, volunteers arrived at the laboratory after a 3-h fast and after abstaining from alcohol, caffeine, and nicotine for at least 3 h. These measures were implemented to minimize external physiological noise and ensure that the recorded HRV reflected the participant’s baseline autonomic state.

### 2.2. Anthropometric Measurements

Participants received a thorough orientation to the testing procedures and time requirements before the assessment. Measurements were obtained while participants wore light clothing and were barefoot, without any extraneous items. Body mass (kg) and height (cm) were recorded using a certified, calibrated mechanical scale and a stadiometer (Filizola, São Paulo, Brazil). Body mass index (BMI) was calculated as weight in kilograms divided by height in meters squared (kg/m^2^) [14].

Circumferential measurements were obtained using a flexible, non-elastic tape measure (Cescorf, Porto Alegre, Brazil) with an accuracy of 0.1 cm. WC was measured at the narrowest part of the torso, between the lower rib cage and the iliac crest. Abdominal circumference (AW) was measured at the midpoint between the lower rib and the iliac crest. Hip circumference was measured at the widest point of the gluteal region. The waist-to-hip ratio (WHR) was then calculated. The tape was positioned horizontally without compressing the skin, and the measurement was recorded at the end of a gentle exhalation. To ensure reliability, the same experienced evaluator performed all measurements three times, and the mean values were used for statistical analysis [15].

### 2.3. Body Composition Assessment

Relative body fat percentage was estimated by bioelectrical impedance analysis (BIA) using the Omron HBF-514C (Omron Healthcare, Kyoto, Japan). The device operated at a single frequency of 50 kHz with a constant alternating current of 500 mA. The assessment was performed in accordance with the standardized bioimpedance guidelines established by the National Institute of Health Technology Assessment Statement [16] to ensure accuracy and reproducibility.

### 2.4. Blood Pressure Assessment

Resting blood pressure (BP) was measured on the left arm using an automated digital oscillometric device (BP7100, Omron Healthcare, Kyoto, Japan). Participants remained seated in a quiet setting for at least 5 min before the assessment. Hypertension was defined as sustained systolic blood pressure (SBP) ≥ 140 mmHg and/or diastolic blood pressure (DBP) ≥ 90 mmHg. If the difference between the first two readings exceeded 5 mmHg, a third measurement was obtained to determine the final BP value [17].

### 2.5. Heart Rate Variability

Resting cardiac autonomic modulation was assessed in a strictly controlled environment to ensure reliable data and stable baselines. Recordings were performed in a temperature-controlled room (22–24 °C) with dim lighting and rigorous noise attenuation. After the BP assessment, participants underwent an additional stabilization period before the 10-min electrocardiogram (ECG) recording began. During this time, participants remained seated and were instructed to breathe quietly and naturally.

Myocardial electrical activity was recorded using a portable, wireless, single-lead ECG system developed by the Biomedical Engineering Program at COPPE/UFRJ in Rio de Janeiro, Brazil. Two surface electrodes were placed on the chest in a modified lead-I configuration. The system uses a BMD101 System-on-a-Chip (NeuroSky, San Jose, CA, USA) for signal acquisition. It includes an integrated analog-to-digital converter and operates at a sampling frequency of 512 Hz. To ensure high signal fidelity and reduce environmental interference, the system incorporates a second-order 60 Hz notch filter and a 100 Hz low-pass filter [18]. Raw ECG data were transmitted via Bluetooth to a computer running the ECG workstation software (version 2.0, COPPE/UFRJ).

The ECG signals were processed using the ECG workstation’s native algorithm, which uses reverse-search derivative filters to detect the QRS complex and accurately identify R-peaks. To ensure the integrity of the HRV analysis, a rigorous artifact-correction protocol was applied. R–R intervals that deviated by more than 20% from the moving average of adjacent intervals were classified as ectopic beats or artifacts and excluded from the dataset [19]. These intervals were then excluded from the HRV analysis. The following time-domain indices were calculated to characterize autonomic tone: (1) the standard deviation of all normal R–R intervals (SDNN), representing total variability; (2) the root mean square of successive R–R interval differences (RMSSD); and (3) the percentage of successive R–R intervals differing by more than 50 ms (pNN50) [20]. Additionally, the cardiac deceleration ratio (CDR) and cardiac acceleration ratio (CAR) were calculated to assess the balance between sympathetic and parasympathetic modulation [21].

### 2.6. Statistical Analysis

Statistical processing and predictive modeling were performed using Python (version 3.11) and the following libraries: Pandas, NumPy, SciPy, Scikit-Learn, Statsmodels, and Pingouin. Descriptive data are expressed as the mean ± standard deviation (SD) and 95% confidence interval (CI).

#### 2.6.1. Preliminary Analysis and Inter-Group Comparisons

We evaluated the normality of the data distribution using the Shapiro–Wilk test and the homogeneity of variances using Levene’s test. An independent samples t-test was used to compare the two age cohorts (30–49 years vs. 50–69 years) for variables that satisfied the parametric assumptions. For variables that did not meet the assumptions of normality or homogeneity, the nonparametric Mann–Whitney U test was used. The effect size (d) was calculated using Cohen’s formula to quantify the magnitude of differences between groups and was interpreted as small (d = 0.2), medium (d = 0.5), or large (d = 0.8).

#### 2.6.2. Logistic Modeling

To address the mathematical dependence of time-domain HRV indices on mean heart rate (HR), a phenomenon known as the circularity problem, vagal modulation was isolated by normalizing the RMSSD to the mean R–R interval (RMSSD_adj = RMSSD/RRmean); both the numerator and denominator are measured in milliseconds. This procedure, recommended by Van den Berg et al. [22], Monfredi et al. [23], effectively isolates intrinsic vagal modulation from the constraining influence of the prevailing HR. Using this dimensionless ratio ensures that the identified phenotypes reflect true physiological differences in autonomic control rather than mathematical artifacts arising from cardiac cycle duration.

In our cohort, the suppressed vagal phenotype was defined as the lowest tertile of RMSSD_adj, corresponding to a cutoff value of 0.0232. Values below this threshold indicate reduced autonomic reserve in this sample. From a clinical perspective, an RMSSD below 20 ms is a recognized marker of increased allostatic load and reduced vagal brake capacity [24]. In our study, the ratio threshold of 0.0232 corresponds to the physiological equivalent of this suppressed raw value and offers a more accurate assessment by accounting for individual variations in resting HR.

Multivariate logistic regression was performed using the binary outcome (RMSSD_adj ≤ 0.0232) to identify significant morphological predictors. To ensure the model’s validity and avoid mathematical circularity, resting HR was excluded as a predictor because it is part of the dependent variable’s calculation. To reduce multicollinearity and prevent model overparameterization, we excluded individual anthropometric components (body mass, height, WC, and hip circumference). Instead, we retained the composite indices BMI and WHR, which effectively reflect overall adiposity and regional fat distribution, respectively.

The final modeling pipeline focused on nine key anthropometric and hemodynamic predictors. To address potential high dimensionality and identify the most reliable predictors, we applied the Least Absolute Shrinkage and Selection Operator (LASSO) for feature selection. The stability of the features selected by LASSO was assessed using the variance inflation factor (VIF), with a threshold of <5 required for inclusion in the final multivariate model. Odds ratios (ORs) and 95% confidence intervals (CIs) were then calculated to quantify the independent association between each morphological associate and the suppressed vagal phenotype.

#### 2.6.3. Validation and Model Performance

Model robustness was ensured using a leave-one-out cross-validation (LOOCV) scheme, which is highly reliable for small sample sizes. To assess the stability and generalizability of the results, we performed bootstrapping with 1000 iterations to generate mean values and 95% CIs for the following metrics: area under the curve (AUC–ROC), sensitivity (recall), specificity, precision, and F1 score. In addition, a calibration curve was generated to evaluate the agreement between predicted probabilities and observed outcomes, thus confirming model reliability.

#### 2.6.4. Development of the Clinical Index

We developed the cardiometabolic–autonomic overload index (ISCA) to integrate the morphological and hemodynamic burden into a single clinical metric. The components of this index, AW, visceral fat, SBP, and WHR, were selected based on the results of LASSO regularization, which identified these components as the most robust correlates of the suppressed vagal phenotype in this dataset.

Using a data-driven approach, the ISCA identifies the variables with the strongest association with autonomic suppression in this population. A cumulative Z-score was calculated for each participant by summing the Z-scores of the four variables. This composite score, representing cumulative physiological burden, was categorized into terciles of ISCA association (low, moderate, and high) and analyzed in relation to HRV parameters using a one-way ANOVA or a Kruskal–Wallis test, followed by Dunn’s post hoc test where appropriate. The significance level was set at α < 0.05 for all procedures.

Each component was given equal weight to ensure a balanced representation of the overall physiological burden and to support clinical interpretability without implying a hierarchy among the selected markers. We acknowledge that, because these components were identified through exploratory analysis in this sample, the ISCA is specific to this sample and requires external validation in independent cohorts.

## 3. Results

The comparative analysis of the two age cohorts (30–49 and 50–69 years) is summarized in Table 1. Most anthropometric parameters, including body mass, BMI, and visceral fat, remained stable between the groups (*p* > 0.05). However, the older cohort had a significantly higher WHR (0.80 ± 0.06 vs. 0.77 ± 0.06; *p* = 0.0159, Cohen’s d = 0.570), indicating a shift toward central adiposity with advancing age. Resting HR was significantly lower in the 50–69 age group (73.68 ± 10.10 bpm vs. 80.41 ± 10.70; *p* = 0.0066, d = 0.646).

In terms of autonomic modulation, the older group showed significantly lower total variability, as indicated by lower SDNN values (35.39 ± 19.28 ms vs. 39.16 ± 15.88 ms; *p* = 0.0419). However, no significant differences in intrinsic vagal modulation (RMSSD) were found between the 30–49 and 50–69 age groups (*p* = 0.261). In contrast, a significant difference in RMSSD was observed when participants were stratified by the ISCA index (*p* = 0.022), with higher morphological load associated with lower intrinsic vagal levels.

We used a multivariate logistic regression model to identify morphological features associated with a suppressed vagal phenotype. For this analysis, we used the lower tertile of heart rate-corrected RMSSD (RMSSD_adj ≤ 0.0232). This normalization procedure effectively isolated intrinsic parasympathetic modulation from the mathematical influence of the prevailing HR. The selection of morphological and hemodynamic predictors followed a rigorous three-step filtering process to ensure model parsimony and eliminate redundancy. (a) Initially, nine variables were considered: chronological age, BMI, AW, WHR, relative body fat percentage, SBP, DBP, CDR, and CAR. (b) The remaining six primary predictors were entered into the LASSO for feature selection. LASSO performed regularization by shrinking the coefficients of less influential variables to zero, thus identifying the most robust correlates of suppressed vagal modulation. (c) The features retained by the LASSO procedure were included in the final multivariate logistic regression to determine ORs. As shown in Table 2, the final model consisted of six independent predictors (WHR, AW, SBP, DBP, relative body fat, and BMI).

Although age was included in the LASSO selection pool, it was not retained as a significant independent predictor in the final model. This supports our primary hypothesis that morphological phenotypes, rather than chronological age, are the primary correlates of autonomic status in this population.

The multivariate logistic regression model identified WHR as the strongest independent predictor of the vagal association phenotype (lower RMSSD adjusted by tertile). As shown in Table 2, each one-unit increase in WHR was associated with more than a twofold increase in the odds of suppressed intrinsic vagal tone (OR = 2.17, 95% CI: 1.15–4.09, *p* = 0.0161). The multicollinearity assessment showed that most features had VIFs below 5, indicating model stability. Although BMI had a slightly higher VIF (6.66), it was not statistically significant in the multivariate context (*p* = 0.33). This further underscores that the topographical distribution of fat (WHR) is a stronger predictor than overall body mass. Other markers, such as AW and visceral fat percentage, showed no significant independent association with the vagal association phenotype after adjustment for WHR (*p* > 0.05).

**Figure 1:**
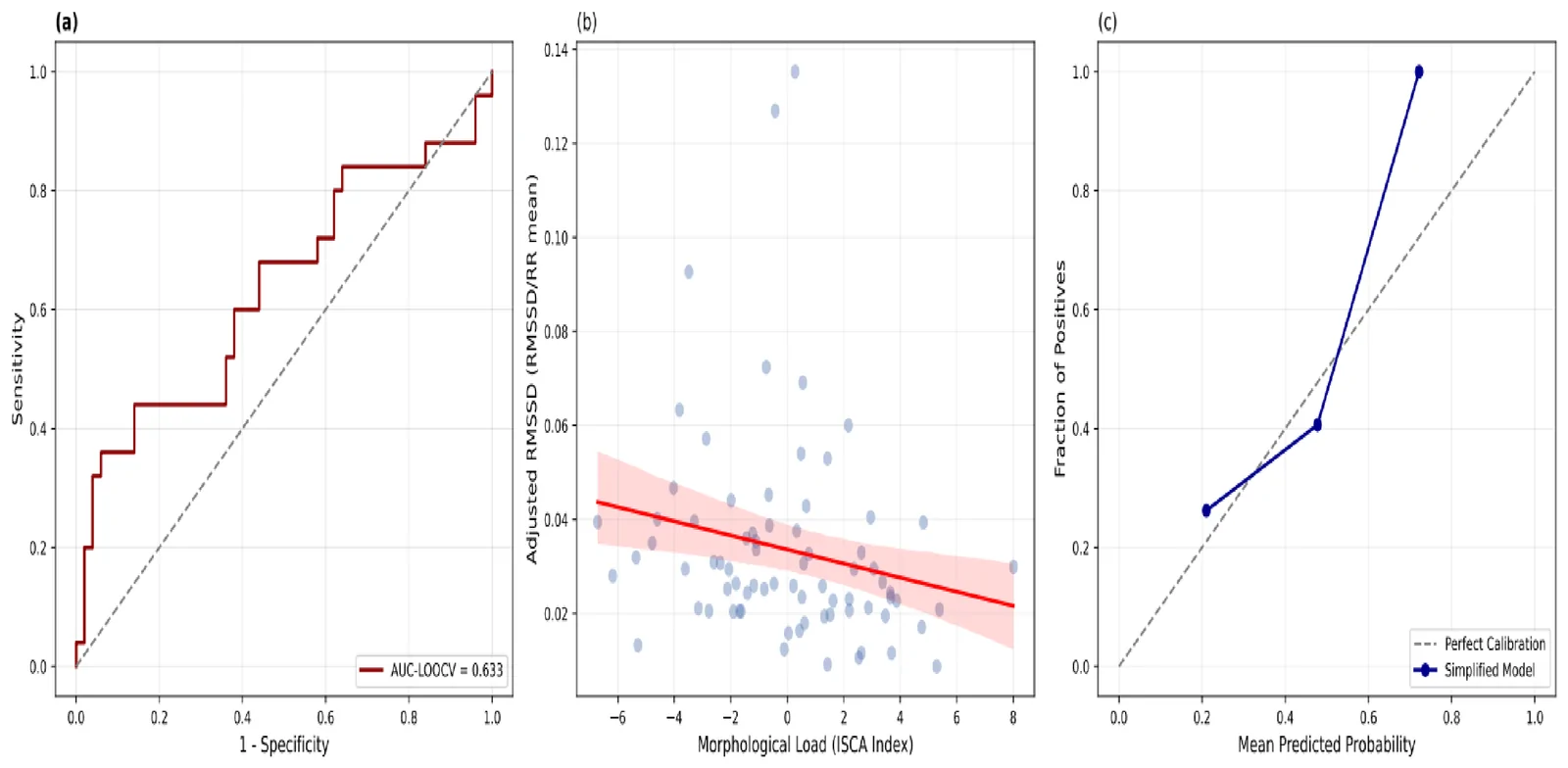
Machine learning validation and clinical application of the autonomic association model. (**a**) Receiver operating characteristic (ROC) curve: Demonstrates the model’s discriminative ability to identify the vagal association phenotype using leave-one-out cross-validation. (**b**) Clinical ISCA index regression: A linear regression analysis demonstrating a significant dose–response relationship between the cumulative ISCA index (Z-score of combined association factors) and vagal tone (RMSSD_adj). The shaded area represents the 95% confidence interval, indicating that greater morphological and hemodynamic overload consistently results in reduced autonomic resilience. (**c**) Calibration Curve: Demonstrates the model’s reliability by comparing the predicted probabilities of autonomic association with the observed proportions and indicating good agreement.

We evaluated the predictive performance of the simplified machine learning model, which uses age and WHR as its primary features, through LOOCV. We then confirmed the model’s performance with 1000 bootstrap iterations to ensure statistical stability (Figure 1a). The model showed moderate discriminatory ability, with a validated area under the curve (AUC) of 0.633 (95% confidence interval [CI]: 0.490–0.771).

In line with its role as a confirmatory clinical tool, the model demonstrated exceptionally high specificity (0.900; 95% CI: 0.814–0.980), highlighting its ability to accurately identify women with preserved autonomic resilience. However, sensitivity (recall) was lower (0.360; 95% CI: 0.179–0.571), suggesting that although morphological phenotypes are strong indicators of autonomic impairment, they do not capture all cases of suppressed vagal tone in this population.

Stratifying the cohort by the ISCA revealed a significant dose–response relationship with intrinsic HRV indices. The Kruskal–Wallis test confirmed that higher levels of cumulative morphological burden were associated with progressive reductions in RMSSD_adj (*p* = 0.0224) and SDNN_adj (*p* = 0.0381), as well as significant changes in the cardiac acceleration ratio (CAR; *p* = 0.0272).

Figure 1b shows the stepwise suppression of intrinsic vagal modulation resulting from the synergistic interaction between central adiposity and hemodynamic load. Notably, the high-overload ISCA phenotype exhibited intrinsic vagal tone that was 44.6% lower than that of the low-overload group. These profound functional differences occurred despite only marginal variations in chronological age between the strata, reinforcing the idea that morphological phenotypes are better indicators of autonomic status than birth year.

The clinical reliability of these associations was further supported by the reliability diagram (Figure 1c), which showed strong agreement between the mean predicted probabilities of the suppressed vagal phenotype and the observed outcomes. The calibration curve demonstrates a consistent upward trend, indicating that higher predicted probabilities successfully identified a larger proportion of individuals with autonomic impairment. Although the model tended to slightly underestimate the probability of impairment at higher risk levels, the overall calibration confirms the clinical utility and robustness of morphological markers for characterizing autonomic status in this population.

The ISCA was developed as a synthetic metric that characterizes the cumulative morphological and hemodynamic burden. The components of this index, AW, SBP, and WHR, were selected based on the LASSO regularization procedure, which identified these features as the most robust associates of the suppressed vagal phenotype in this cohort. Although AW did not reach statistical significance as an independent predictor in the final multivariate logistic regression model (Table 2), it was retained within the ISCA framework. This decision was based on its consistent selection by the machine learning pipeline. This suggests that visceral fat is a key constituent of the morphological-hemodynamic fingerprint associated with autonomic impairment. This is true even though its individual statistical contribution was attenuated when modeled alongside other highly collinear variables.

As shown in Table 3, the transition from a low- to a high-association phenotype was marked by a significant increase in central adiposity and hemodynamic stress. Women in the high-association category showed a substantial increase in AW, averaging 20.8 cm (from an average of 82.2 cm in the low-association group to 103.0 cm in the high-association group). These morphological changes were accompanied by an increased hemodynamic load, reflected in an 8.3 bpm increase in resting HR (85.10 bpm in the high group versus 76.80 bpm in the low group). Therefore, the high-association phenotype exhibited an RMSSD of only 18.70 ms, representing a 44.6% lower vagal tone compared to the low-association group (33.78 ms; *p* < 0.05).

**Figure 2:**
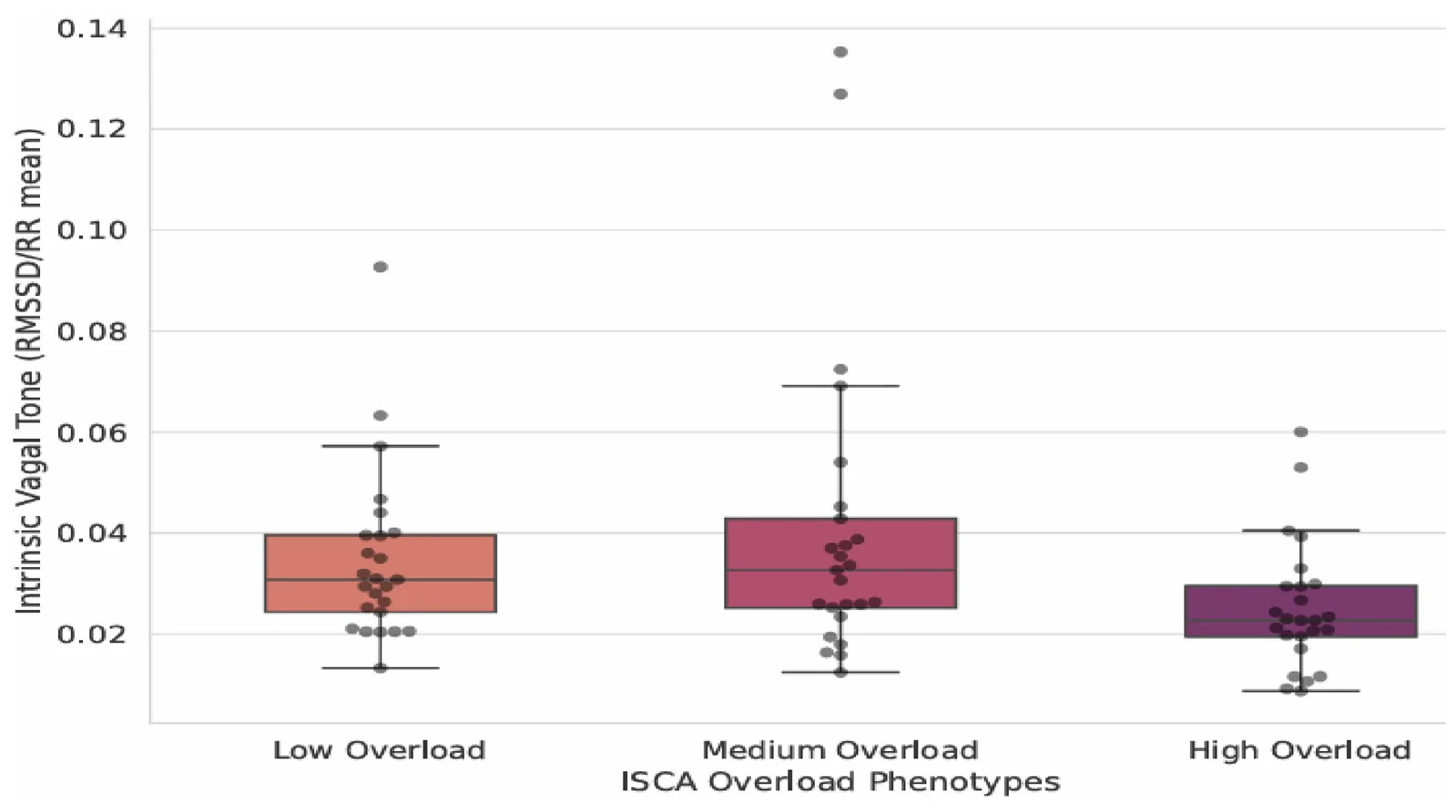
Dose–response relationship of the ISCA Index: boxplots combined with individual data distributions illustrate the progressive decline in intrinsic vagal tone (RMSSD_adj) across the association categories of the cardiometabolic–autonomic overload index (ISCA). Horizontal lines represent the median, and the boxes indicate the interquartile range. Statistically significant suppression of autonomic resilience is observed as morphological burden increases from low to high association (*p* < 0.05).

Using the ISCA, we stratified the cohort into low, medium, and high morphological burden categories. This revealed a significant dose–response relationship with intrinsic vagal modulation. As shown in Figure 2, intrinsic vagal tone (RMSSD_adj) progressively and stepwise decreased as cumulative morphological burden increased. Women in the high-overload group exhibited markedly lower autonomic resilience than those in the low-overload group. The boxplots and integrated individual data distributions in Figure 2 show that this downward trend is statistically significant (*p* < 0.05). This finding highlights that greater central adiposity and hemodynamic load are associated with reduced vagal activity, regardless of chronological age.

Critically, these profound functional differences emerged despite only marginal differences in chronological age between the cohorts (mean age: 48.3 years in the low-association group vs. 51.2 years in the high-association group). Furthermore, while the adjusted RMSSD declined sharply across categories, the resting HR remained relatively stable as the association increased from moderate to high. This suggests that the observed decrease in autonomic vagal modulation is linked to the functional consequences of morphological burden rather than being a mathematical byproduct of HR acceleration. The dissociation between birth year and physiological status reinforces the idea that the ISCA index characterizes the morphological–hemodynamic burden. Chronological age was not maintained as an independent predictor in this model. This offers a more accurate measure for stratifying cardiometabolic associations in women.

## 4. Discussion

This study examined the complex relationship between morphological phenotypes and cardiac autonomic modulation in women. Our primary finding is that autonomic resilience is more closely associated with an individual’s morphophysiological phenotype, as represented by the ISCA index, than with chronological age. Although the ISCA overload score did not differ significantly between the age groups, our results support the hypothesis that the cardiac autonomic profile is more strongly linked to morphological burden than to the mere passage of time.

### 4.1. The Primacy of Central Adiposity: WHR vs. BMI

Our results reinforce the growing consensus in kinesiological research that indices of central adiposity are more reliable indicators of reduced HRV and sympathovagal imbalance than general markers such as BMI [25]. In our model, the WHR was the only significant independent predictor of lower intrinsic vagal modulation. This finding supports the view that trunk and visceral fat depots are metabolically active and linked to systemic inflammation and altered baroreflex sensitivity [26].

Although BMI is a widely used proxy for adiposity, it does not differentiate between fat mass and lean mass. In our cohort, the statistical significance of BMI declined after WHR was included in the multivariate model. This suggests that android fat distribution, characterized by an elevated WHR, is the main morphological correlate of reduced vagal modulation. This morpho-autonomic profile is likely associated with the pro-inflammatory environment of visceral fat, which is linked to increased sympathetic outflow and reduced parasympathetic modulation [27].

### 4.2. Addressing Heart Rate Dependence and Biological Age

A major methodological strength of this study was the application of an HR correction for RMSSD. As noted previously, time-domain HRV indices are inherently constrained by HR [22,23]. By normalizing RMSSD to the R–R interval, we were able to isolate the intrinsic vagal component. The finding that WHR remained a significant factor even after this correction suggests that the association between central fat and lower intrinsic vagal modulation is biological rather than mathematical, which is a common pitfall in HRV research [28].

The divergence between chronological age and ISCA index categories is especially relevant during the menopausal transition. Women moving from middle to older age often show visceralization regardless of weight gain [29]. Our data suggest that a 40-year-old woman with a high WHR may have an autonomic profile consistent with a more advanced physiological age. This indicates phenotypic similarity to an older cohort. It represents a functional equivalent of accelerated aging and underscores the autonomic nervous system’s sensitivity to the internal milieu [30].

### 4.3. The Predominance of Central Adiposity over Overall Metrics

Our results support the consensus that central adiposity is a more accurate indicator of autonomic dysfunction than global markers. BMI showed moderate correlations with HRV. However, measures of central fat distribution, specifically AW and WHR, demonstrated stronger associations with reduced markers of intrinsic vagal modulation. These findings are consistent with the work of König et al., who proposed that visceral fat functions as a metabolically active tissue associated with systemic inflammation and altered baroreflex sensitivity [31,32].

In our cohort, the high-association phenotype was characterized by an AW 20 cm greater and an RMSSD 44.6% lower than those of the low-association group. This morpho-autonomic state is likely linked to the metabolic activity of visceral adipose tissue, which is associated with a pro-inflammatory environment and elevated leptin levels. These factors are well-established correlates of increased sympathetic activity and reduced vagal modulation. Our results support the assertion that the nonlinear complexity of the autonomic nervous system is better characterized through integrated morphological modeling than through isolated anthropometric variables [33].

### 4.4. The Menopausal Transition and the Biological Age Hypothesis

One of the main conclusions of this study is that central adiposity, in particular, is more strongly associated with autonomic modulation than chronological age. This claim is supported not only by the absence of significant differences in RMSSD between age cohorts, but also by the results of our predictive modeling.

When chronological age was included in the initial LASSO selection pool alongside morphological variables, it was not retained as a significant independent predictor. In contrast, markers of central adiposity remained significantly associated with the vagal phenotype even after adjustment for other variables in the multivariate regression analysis. These results suggest that, for women aged 30–69, the degree of visceralization and hemodynamic load may be better indicators of autonomic status than birth year alone. However, given the cross-sectional design, these findings should be interpreted cautiously because they indicate an association rather than a causal relationship between morphology and autonomic decline. The transition into menopause is a pivotal physiological milestone associated with changes in cardiometabolic profiles. The absence of significant differences in ISCA scores across age groups may be related to the loss of estrogen’s cardioprotective effects. The decline in estrogen during perimenopause and its deficiency during the postmenopausal period primarily contribute to visceralization, shifting fat distribution from gynoid (peripheral) to android (central) patterns [34].

This morphological shift is associated with reduced vagal modulation. This biological transition likely explains why suppressed vagal phenotypes were observed in the younger cohort (30–49 years old). Women in the late perimenopausal phase may display a cardiovascular profile more typically associated with older age. In contrast, postmenopausal women (50–69 years old) with favorable morphological profiles demonstrate preserved autonomic resilience. Thus, the ISCA index effectively captures the hallmarks of the menopausal transition (cumulative visceral stress and hemodynamic demand), indicating that autonomic status is more closely aligned with the hormonal-morphological axis than with chronological age [29].

### 4.5. Model Performance and Clinical Phenotyping

Our model’s high specificity (81.9%) reflects its discriminative performance, making it a robust confirmatory tool. Clinically, this indicates that when a woman presents with an elevated WHR and a high ISCA score, her autonomic vulnerability can be identified with a high degree of certainty. As shown in Table 3, the high-association phenotype is characterized by an increase of nearly 21 cm in AW and a 44.6% decrease in vagal tone, despite minimal differences in age.

From a practical perspective, these findings point to a paradigm shift in kinanthropometric assessment. Incorporating simple, low-cost markers of central fat distribution into routine screenings offers an inexpensive yet effective way to evaluate a patient’s autonomic health. This makes it possible to identify vulnerable phenotypes earlier and implement targeted lifestyle interventions that promote autonomic resilience throughout a woman’s life.

Our ISCA Index revealed a clear dose–response relationship: as cumulative association scores increased, RMSSD values decreased from 33.78 to 18.70 ms. This confirms that, although aging is generally associated with a decline in SDNN, likely owing to intrinsic changes in the sinus node, the vagal protection reflected by RMSSD is susceptible to modification by body composition. This finding is consistent with that of Cvijetic et al. [35], who noted that physical markers, such as muscle mass and fat distribution, can offset or accelerate the negative interactions between aging and HRV. RMSSD was chosen as the primary outcome variable because it is highly sensitive to rapid changes in vagal tone and is statistically more robust than other time-domain indices in short-term recordings [36].

A major strength of this study was the use of a portable, wireless ECG system developed at COPPE/UFRJ, which enabled high-fidelity signal acquisition (512 Hz) in a controlled setting. In addition, combining LASSO variable selection with LOOCV-bootstrap validation addresses a common limitation in kinanthropometric research: overfitting in small samples. We established a validated framework for identifying women with lower intrinsic autonomic vagal modulation using noninvasive measurements, achieving an AUC of 0.633 even after HR correction.

### 4.6. Limitations and Future Directions

Despite the strong validation of our findings, several limitations should be acknowledged. First, our recruitment strategy limits the generalizability of the results. Participants were randomly selected when they first enrolled at a local fitness center, which may have introduced a healthy volunteer effect. People who begin a fitness program typically have greater health motivation and different physiological profiles than the general sedentary population [37]. This selection bias is particularly relevant because better aerobic fitness is associated with enhanced post-exercise vagal re-entry. However, it may not fully counteract the inherent decline in resting HRV associated with biological aging [38].

Furthermore, our model’s predictive performance underscores its utility as a confirmatory rather than comprehensive tool. Although the model achieved high specificity (0.900), the moderate area under the curve (AUC = 0.633) and sensitivity (0.360) indicate that morphological load is a significant, but not the only, determinant of autonomic status. These metrics further suggest that, although the ISCA index effectively identifies women with preserved autonomic resilience, other physiological, genetic, or lifestyle factors not examined in this study likely also influence vagal modulation.

Methodologically, our exclusive focus on time-domain HRV indices was intentional to ensure data integrity. We excluded frequency-domain parameters, such as high-frequency (HF) and low-frequency (LF) power, because respiratory rate was neither paced nor monitored. Under spontaneous breathing conditions, fluctuations in respiratory frequency can substantially distort spectral results by shifting the respiratory sinus arrhythmia (RSA) peak [39]. In contrast, RMSSD is recognized for its robustness and lower sensitivity to respiratory variations, providing a stable measure of vagal tone. Additionally, we applied an HR correction to RMSSD to address the mathematical dependency identified by Barbosa et al. [40].

A key consideration in our stratification is the role of the menopausal transition. Because the older group was postmenopausal by design and the younger group was in the early follicular phase, menopausal status is inherently correlated with age group and central adiposity. Estrogen withdrawal independently promotes central fat redistribution and alters cardiac autonomic modulation. Therefore, the associations described here may partly reflect parallel consequences of the menopausal transition rather than a direct link between adiposity and autonomic function. Because menopausal status was not confirmed through hormonal profiling, our study design cannot fully distinguish these concurrent biological processes.

Future research should use longitudinal designs to monitor how changes in morphological phenotypes, such as targeted weight loss or increases in skeletal muscle mass, affect the recovery of the vagal brake. It is also essential to validate the ISCA index in more diverse populations, including sedentary individuals and those with subclinical cardiometabolic conditions. Incorporating objective measures of physical activity, such as accelerometry, together with comprehensive hormonal profiling, is crucial for advancing our understanding of the interactions between morphological structure and autonomic resilience across the female lifespan.

## 5. Conclusions

This study indicates that morphological structure, specifically central adiposity, was associated with suppressed vagal modulation, whereas chronological age was not retained as an independent predictor in the present model. The association between central fat and lower intrinsic vagal modulation remained significant after correcting for the mathematical dependency of HRV on heart rate, suggesting an underlying physiological link. The development of the ISCA index provides a framework for identifying autonomic phenotypes characterized by cumulative morphological burden. These findings highlight that central adiposity distribution may reflect attenuated autonomic resilience regardless of birth year, emphasizing the need for personalized kinanthropometric assessments that prioritize biological and functional status over traditional age-based metrics.

## Figures and Tables

**Table 1:** Anthropometric, hemodynamic, and autonomic characteristics of the study population, stratified by age group.

Variables	Total Sample (*N* = 75)	30–49 Years (*n* = 38)	50–69 Years (*n* = 37)	*p*-Value	Cohen’s *d*
Age (years)	49.3 ± 11.2	39.4 ± 5.8	59.2 ± 5.9	<0.001 *	3.38
Body mass (kg)	74.9 ± 14.7	74.9 ± 16.4	74.9 ± 12.9	0.995	0.001
Height (cm)	163.0 ± 5.9	164.0 ± 5.3	162.0 ± 6.3	0.137	0.346
BMI (kg/m^2^)	28.1 ± 5.3	27.8 ± 6.12	28.5 ± 4.5	0.586	0.126
Waist circumference (cm)	83.9 ± 10.5	81.8 ± 11.2	86.1 ± 9.3	0.073	0.420
Abdominal circumference (cm)	92.0 ± 12.3	89.4 ± 12.8	94.8 ± 11.3	0.056	0.447
Hip circumference (cm)	107.1 ± 10.8	106.6 ± 11.6	107.6 ± 10.0	0.510 †	0.093
Waist-to-hip ratio	0.78 ± 0.06	0.77 ± 0.06	0.80 ± 0.06	0.015 *	0.570
Relative body fat percentage	40.2 ± 8.2	39.0 ± 9.4	41.5 ± 6.4	0.184	0.310
Systolic BP (mmHg)	116.9 ± 17.2	114.5 ± 14.0	119.4 ± 20.0	0.432 †	0.278
Diastolic BP (mmHg)	76.1 ± 10.7	77.3 ± 10.5	75.0 ± 10.9	0.352	0.216
Resting heart rate (bpm)	77.1 ± 10.9	80.4 ± 10.7	73.6 ± 10.1	0.006 *	0.646
SDNN (ms)	37.3 ± 17.6	39.1 ± 15.8	35.3 ± 19.2	0.041 *	0.213
RMSSD (ms)	27.8 ± 21.7	27.4 ± 15.9	28.17 ± 26.8	0.261	0.032
pNN50 (%)	6.3 ± 11.0	6.0 ± 7.9	6.6 ± 13.6	0.324	0.056
CDR	20.0 ± 14.3	20.0 ± 10.1	20.0 ± 17.7	0.222	0.004
CAR	20.5 ± 12.5	20.3 ± 9.3	20.7 ± 15.3	0.426	0.029

Notes: Data are presented as the mean ± standard deviation. BMI: body mass index; BP: blood pressure; SDNN: standard deviation of all normal R–R intervals; RMSSD: root mean square of successive differences; pNN50: percentage of successive R–R intervals differing by >50 ms; CDR: cardiac deceleration ratio; CAR: cardiac acceleration ratio. * Indicates a statistically significant difference (*p* < 0.05). † Analyzed using the Mann–Whitney U test (nonparametric); all other analyses used the independent samples t-test.

**Table 2:** Multivariate logistic regression and machine-learning validation for predicting the vagal association phenotype.

Feature Selected by LASSO	VIF	Standard Error	Odds Ratio (OR)	95% CI for OR	*p*-Value
Intercept (constant term)	-	0.12	0.45	0.26–0.77	0.0038 *
Waist-to-hip ratio	1.35	0.70	2.17	1.15–4.09	0.0161 *
Abdominal circumference	4.62	0.52	0.91	0.29–2.81	0.8758
Systolic blood pressure	2.34	0.24	0.60	0.26–1.35	0.2189
Diastolic blood pressure	2.28	0.57	1.40	0.62–3.13	0.4071
Relative body fat	3.98	0.49	0.84	0.26–2.65	0.7723
Body mass index	6.66	1.34	1.94	0.50–7.51	0.3377

Notes: LASSO: least absolute shrinkage and selection operator; VIF: variance inflation factor (values below 5 indicate low multicollinearity). The target variable, vagal association, is defined as RMSSD_adj ≤ 0.0232. * indicates a statistically significant predictor in the multivariate model.

**Table 3:** Mean values of morphological, hemodynamic, and autonomic variables stratified by clinical ISCA index levels.

ISCA Association Category	Resting HR	Abdominal Circ.	Relative Body Fat	Systolic BP	Waist-to-Hip Ratio	RMSSD
Low association	76.80	82.20	32.96	103.80	0.74	33.78
Moderate association	76.88	91.16	40.51	118.40	0.79	31.16
High association	85.10	103.08	47.44	128.92	0.82	18.70

Notes: HR: heart rate; Abdominal Circ.: abdominal circumference; BP: blood pressure; RMSSD: root mean square of successive differences. The ISCA index (cardiometabolic–autonomic overload) is a cumulative z-score of morphological and hemodynamic variables.

## Data Availability

The datasets generated and analyzed in the current study are not publicly available due to privacy and ethical restrictions related to the sensitive nature of participants' clinical, anthropometric, and physiological data. However, anonymized data supporting the findings of this study are available from the corresponding author upon reasonable request.
